# The complete mitochondrial genome characterization of *Tridentiger obscurus* (Gobiiformes: Gobiidae) and phylogenetic analyses of Gobionellinae

**DOI:** 10.1080/23802359.2017.1375874

**Published:** 2017-09-12

**Authors:** Li Gong, Xun Du, Zhen-Ming Lü, Li-Qin Liu

**Affiliations:** a National Engineering Laboratory of Marine Germplasm Resources Exploration and Utilization, Zhejiang Ocean University, Zhoushan, China;; b National Engineering Research Center for Facilitated Marine Aquaculture, Marine Science and Technology College, Zhejiang Ocean University, Zhoushan, China

**Keywords:** Dusky tripletooth goby, mitogenome, phylogenetic analyse

## Abstract

The dusky tripletooth goby, *Tridentiger obscurus*, is a good model organism for the small in size and reaching maturity within a single year. Previous studies mainly focused on the annual reproductive cycle, social behavior and life history, but little information is available of the mitochondrial genome and phylogenetic evolution of this gobioid fish. In this article, we described the complete mitogenome of *T. obscurus* and reconstructed the phylogenetic relationship of the relative species of Gobionellinae. The genome is 16,501 bp in length including 13 protein-coding, two ribosomal RNA, 22 transfer RNA genes, as well as a putative control region and an L-strand replication origin. The overall base composition is 28.1%, 27.0%, 28.0% and 16.9% for A, T, C and G, respectively. This result is expected for better understanding the systematic evolution of the genus *Tridentiger* and further phylogenetic study of Gobiiformes.

The dusky tripletooth goby, *Tridentiger obscurus*, is a small fish, commonly found in both fresh and brakish waters of East China, Japan and Korea (Kim et al. 2005). This species is a good model organism for the small in size and reaching maturity within a single year (Kaneko and Hanyu 1985). Previous studies mainly focused on the annual reproductive cycle (Kaneko and Hanyu 1985), social behaviour (Kishi 1979) and life history (Nakamura 1942), but little information is available of the mitochondrial genome and phylogenetic evolution of this gobioid fish. Here, we described the complete mitogenome of *T. obscurus*, and reconstructed the phylogenetic relationship of the relative species of Gobionellinae, expecting for better understanding the systematic evolution of the genus *Tridentiger* and further phylogenetic study of Gobiiformes.

The specimen was collected from Fukushima, Japan (37.7512°N; 140.7055°E) and was stored in 95% ethanol with accession number 20141024FA02. The complete mitogenome of *T. obscurus* is 16,501 bp in length (GenBank accession MF663787), containing 13 protein-coding genes, 22 tRNA genes, two rRNA genes, as well as a control region (CR) and an L-strand replication origin (O_L_). Gene arrangement and contents are identical to those of typical teleostean mtDNA (Ponce et al. 2008; Liu et al. 2013; Gong et al. 2016). Most of these genes are encoded by the H-strand, except the *ND6* and eight tRNA genes. The overall base composition was 28.1%, 27.0%, 28.0% and 16.9% for A, T, C and G, respectively. All protein-coding genes use the initiation codon ATG except *COI* gene, which begins with GTG. In addition, most protein-coding genes use TAA or TAG as stop codon, except *ND4* uses AGA, *COII*, *COIII* and *Cyt b* genes use an incomplete stop codon T. The 13 protein-coding genes encoded 3801 amino acids and the most frequently used amino acid is Leucine (17.42%), while Cysteine acid (0.67%) is the least one.

The two rRNA genes (12S and 16S) are generally isolated by *tRNA*-*Val*, located between *tRNA*-*Phe* and *tRNA*-*Leu*. The 22 tRNA genes are interspersed between rRNAs and protein-coding genes, with sizes ranging from 66 bp (*tRNA*-*Cys*) to 75 bp (*tRNA*-*Leu* and *tRNA*-*Lys*). The 51-bp O_L_ was located between *tRNA*-*Asn* and *tRNA*-*Cys* genes and has the potential to fold into a stable stem-loop structure (Zhang et al. 2014; Gong et al. 2017). The entire control region was bounded by *tRNA*-*Pro* and *tRNA*-*Phe* with a length of 851 bp. The symbolic structures of the CR are observed as in other fishes such as the TAS-cTAS, central conserved sequence blocks (CSB-F, D, B, A), CSB 2-3, G-box (GTGGGGG) and a pyrimidine tract (Lee et al. 1995; Guo et al. 2003; Gong et al. 2015).

Based on the 13 protein-coding genes of each mitogenome from 22 Gobionellinae species, with *Pseudogobius javanicus* and *P. taijiangensis* as outgroup, a maximum likelihood phylogeny tree was constructed by using MEGA5 (Tamura et al. 2011). The tree clearly revealed that four *Tridentiger* species first clustered a group, and then formed a sister-group with other Gobionellinae species (Figure 1).

**Figure 1.**
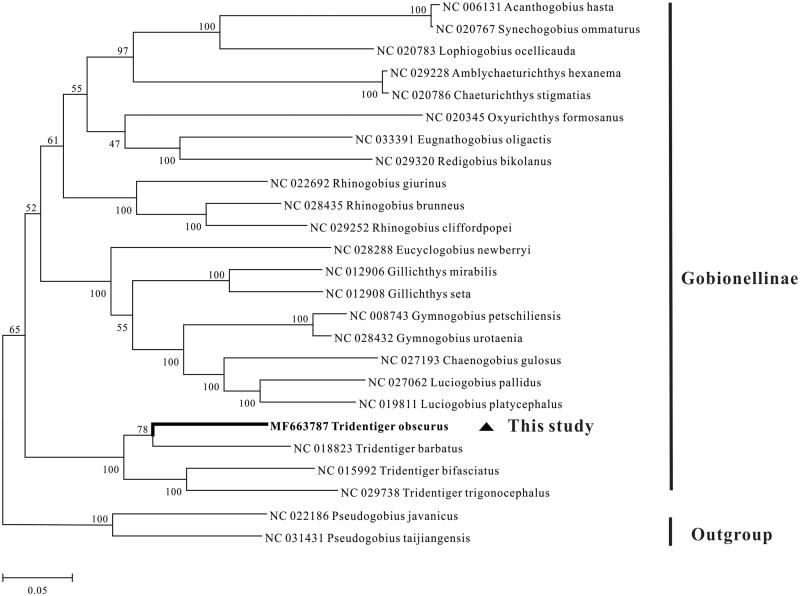
Phylogenetic tree of Salmoninae based on the maximum likelihood (ML) analysis of 13 protein-coding genes. The GTR + I + G model was the most appropriate model based on the Akaike Information Criterion (AIC). The number at each node is the bootstrap probability. The number before the species name is the GenBank accession number.
